# Evaluation of antimicrobial prophylaxis use and rate of surgical site infection in surgical ward of Wachemo University Nigist Eleni Mohammed Memorial Hospital, Southern Ethiopia: prospective cohort study

**DOI:** 10.1186/s12879-019-3895-5

**Published:** 2019-04-02

**Authors:** Bruke B. Billoro, Mengistu H. Nunemo, Seid E. Gelan

**Affiliations:** 1 Department of Pharmacy, College of Medicine and Health Sciences, Wachemo University, Hosanna, Ethiopia; 2 Department of Public Health, College of Medicine and Health Sciences, Wachemo University, Hosanna, Ethiopia; 3 Ethiopian Biotechnology Institute, Addis Ababa, Ethiopia; 4 Department of Clinical Pharmacy, Near East University, Nicosia, Northern Cyprus Cyprus

**Keywords:** Risk factors, Surgical wound infection, Susceptibility, Prospective, Ethiopia

## Abstract

**Background:**

Surgical site infection (SSI) is the foremost infection in the overall patient population, affecting up to 66% of operated patients and with a frequency up to nine times more than in developed countries. This study aimed to determine the rate, associated factors of surgical site infection, and identification of causative agents and their antimicrobial susceptibility in surgical ward of Wachemo University Nigist Eleni Mohamed Memorial Hospital (WUNEMMH), Southern Ethiopia.

**Method:**

Prospective cohort study involving 255 patients who underwent surgical procedure in WUNEMMH from January 1 to September 1, 2017. We extracted data from medical chart, operational and anesthesia note by direct observation and interviewer administered semi-structured questionnaire which was validated. We collected wound specimens and processed it based on standard operating procedure, and disc-diffusion antibiotic susceptibility test was done. Data analysis was done using SPSS version 22.0. Factors significantly associated were identified using logistic regression model at *P*-value < 0.05 and 95%CI.

**Result:**

Forty-two patients (16.5%) developed SSIs. The most causative organism of surgical site infection was *Klebsiella pneumoniae* (60%).Ciprofloxacin and ceftriaxone were sensitive antibiotics. Surgery waiting time more than 7 days [ARR = 2.48 (95%CI(1.28–4.79),*P* = 0.007], Operation time more than 1 h.[ARR = 2.13(95%CI(1.18–3.86),*P* = 0.012], and administering antibiotic before 1 h of operation [ARR = 5.05(95%CI(1.79–14.21),*P* = 0.002], smoking [ARR = 8.01 (95% CI (2.15 29.84),P = 0.002] were independently associated with surgical site infections.

**Conclusion:**

The rate of SSI was relatively high. *Klebsiella pneumoniae* was found to be the most causative agent for SSI. Organisms causing SSI were sensitive to commonly used antimicrobial agents in WUNEMMH.

## Background

A surgical site infection (SSI) is an infection which happens within thirty days at the operative site if no graft is left in place or within ninety (90) days if an insert is left in place after the surgical operation. The surgical procedure determines the category of infection must appear [1].

SSIs are the third common hospital acquired infection which accounts 38% of all nosocomial infections according to center for disease control (CDC’s) national nosocomial infection surveillance system [2].

In countries with limited resources, SSI is the foremost infection in the overall patient population, affecting up to 66% of operated patients and nine times more than in industrialized countries [3].

Increase hospital stays time, readmissions and additional use of antimicrobials which lead to antibiotic-resistant were seen by SSIs [4].On average, SSIs increased postoperative hospital stay by 6.5 to 11 days and 3.57 additional drug uses [2, 4]. SSI increased cost in England from $1,341-10,922 per patient, in Europe € 3,985/day and in the USA $25,546 per infection depending on surgery type and infection severity at institutional as well as national level [5, 6].

Study indicated that nearly 40-60% of SSIs can be prevented with the appropriate use of surgical antimicrobial prophylaxis (AMP) [7, 8]. Approximately 30-50% of antimicrobials used in hospitals are for surgical prophylaxis but 30 -90% of them are used inappropriately [9, 10] which attribute for 16% of SSIs [8].

Antimicrobials are a serious matter and resources are in short supply to prevent rise of resistance and limit the degree of this problem in developing countries. Although pathogens resistivity has increased, unfortunately, no new antimicrobials that can manage this resistance have been introduced and are not expected in the future [11, 12]. World Health organization (WHO) described as resistance to antimicrobials has been a main threat to worldwide public health because there are few antimicrobials available to treat life-threatening infections in some cases [13].

Unnecessary extra costs, potential promotion of microbial resistance selection and a precious resource wastage in health care occur due to inappropriate use of AMP. Studies presented that the cost of inappropriate AMP is approximately 10 times higher than the values expected [14, 15].

Gram-positive cocci and gram-negative bacilli are major causative organisms of SSI as described by multi and single centered studies [16, 17].

Due to inadequate SSI surveillance programs in many of sub-sahara Africa, healthcare centers are unable to get an update on bacteria which is resistant to the antimicrobial [18]. In developing countries tests which would be used to isolate the organism and the correct antibiotic has limited accessibility and affordability [19].

Periodic surveillance and feedback to surgeons on the rate of SSIs and factors significantly associated can reduce up to 50% of SSIs rate according to WHO and other studies [5, 20].

Studies on rates of SSI, factors independently associated with SSI as well as on the causative agents and antibiotics susceptibility test are scarce in Ethiopia.

To our knowledge, studies focusing on SSI causing bacteria and their sensitivity pattern to antibiotics were not done in the study area.

Therefore, our study aimed to show the rate of SSI as well as factors associated with SSIs and characterize the susceptibility patterns of bacterial agents causing SSI at surgical ward department of WUNEMMH.

## Methods

### Study area

Conducted in surgical department of WUNEMMH which is government hospital.

### Study design and period

A prospective cohort study in the surgical ward from January 1, 2017, to September 1, 2017, at WUNEMMH.

### Study population

All patients who admitted for elective and emergency surgical procedures, and who fulfill eligibility criteria during the study period.

### Sample size

Calculated using single population proportion formula with 훼 of 5% and proportion for SSI rate is 19.1 % [21]. By considering 10% contingency, total sample size would be 280 patients.

### Study procedures

Patients who were not willing to participate, receiving antimicrobial during admission or stopped receiving within 48 hours before operation and patients with initial diagnosis suggestive of infection were excluded. Written consent was obtained and interviewer administered semi-structured questionnaire was used for socio-demographic characteristics.

Socio-demographic and patient-related factors were collected from patient’s medical chart. Data on antimicrobials administration after operation and duration of administration were obtained from medication chart and direct observation.

Data were collected by three Nurses (BSc) using pretested data collection tool and the data collection was supervised daily by the research team.

Wound classification was done using Center for Disease Prevention and Control (CDC) criteria for surgical site infections surveillance and WUNEMMH surgeons were responsible for diagnosing surgical patients’ infection

### Specimen collection procedures

Sterile swabs were used for collection of exudates from wounds of patients who develop SSI which transferred at room temperatures to the laboratory within 20 minutes. Chocolate, blood, and Mac-Conkey agar were used for inoculating swabs. We sited chocolate plate with other plates in a candle jam jar and keep warm at 35–37 °C for 24–48 hours. We report organisms by performing gram stain procedure on culture growth. An additional blood agar plate was inoculated anaerobically at 35–37 °C for 48–72 hours.

### Sensitivity pattern of antibiotics

Disk diffusion test was performed for testing sensitivity [22]. They were exposed to gentamicin, erythromycin, ceftriaxone, ciprofoxacillin, chloramphenicol (CAF), cloxacillin, ampicillin, and tetracycline. We did not test for methicillin resistant *Staphylococcus aureus* (MRSA) and gram negatives which are extended-spectrum producers since this was not our study aim.

### Statistical analysis

We used Epi-Data version 3.1 and analyzed using SPSS for window version 20.0. Frequency and percentages were used to describe socio-demographic, surgery-related factors, type of surgery and sensitivity of causative agents. Before running a multivariable binary logistic regression, multicollinearity between the independent variables was checked in linear regression by variance inflation factors (VIF). The model fitness for the variables was evaluated by the Hosmer-Lemeshow goodness of fit test. P-value of less than 0.05 was considered as independently associated in the multivariable analysis.

## Result

### Social demographic factor

Twenty-five patients out of 280 patients were excluded based on the exclusion criteria. The analysis was done on a total of 255 patients. 147 patients (57.65%) were males and more than half patients were from rural area 150(58.8%). Nine patients (3.5%) were obese with BMI > = 30 kg/m2. Twelve patients (4.7%) received blood transfusion preoperatively (Table 1).

**Table 1:** Socio-demographic factors in surgical ward of WUNEMMH from January 1 to September 1, 2017 (*N* = 255)

Variables		Frequency	Percent
Residence	Urban	105	41.2
	Rural	150	58.8
Cigarette smoking	No	192	75.3
	Yes	63	24.7
Gender	Female	108	42.35
	Male	147	57.65
Age categories in a year	1–18	63	24.7
	19–40	111	43.5
	> 40	81	31.8
Obesity	BMI < 30 kg/m2	246	96.5
	BMI > =30 kg/m2	9	3.5
Preoperative blood transfusion	No	243	95.3
	Yes	12	4.7
Surgery waiting time	<=7 days	198	77.65
	>7 days	57	22.35
ASA score	I	66	25.9
	II	171	67
	III	18	7.1

### Surgery-related factor

One hundred forty-four (56.5%) surgical procedures were clean wound and 177(69.4%) surgical procedures were elective. 225 (88.2%) had no history of previous surgical procedure (Table 2).

**Table 2:** Surgical related factors in the surgical ward of WUNEMMH from January 1 to September 1, 2017 (*N* = 255)

Variables	Frequency	Percent
Previous surgery		
Yes	30	11.8
No	225	88.2
Wound class		
Clean	144	56.5
Clean-contaminated	111	43.5
Surgical type		
Elective	177	69.4
Emergency	78	30.6

### Surgery procedure type

Caesarean section, 72(28.2%) was the leading procedure followed by appendectomy, 54(21.2%), head and neck surgery, 32(12.5%) (Table 3).

**Table 3:** Surgical procedure done in the surgical ward of WUNEMMH from January 1 to September 1, 2017 (N=255)

Variables	Frequency	Percent
Caesarean section	72	28.2
Appendectomy	54	21.2
Head and neck surgery	32	12.5
Genitourinary surgery	30	11.8
Hernia repair	28	10.9
Breast surgery	21	8.2
Hepato-biliary surgery	15	5.9
Lipoma excision	3	1.2
Total	255	100.0

#### Rate of SSI

Out of 255 patients, 42(16.5%) patients developed SSIs. Among patients who developed SSIs, 38 (90.47%) patients developed SSIs in hospital.

We followed patients and reviewed charts daily before, during and after operation until the patients were discharged from the hospital and after discharge till 30 days since operation was done

#### Etiological organisms for SSIs

*Klebsiella pneumonia* was found to be the most common for 60.0% of isolates followed by *Staphylococcus aureus* (17.8%), *Escherichia colli* (11.1%) and *Pseudomonas aeruginosa* (11.1%).

#### Sensitivity of causative organisms

Ceftriaxone was used as prophylactic antibiotic in study area. Susceptibility test to identified organisms were done in eight drugs. 35% of them had resistance to organisms which include ampicillin, tetracycline and erythromycin. The remaining 65% of drugs had different sensitivity patterns.

*Klebsiella species* were 88.9% susceptible to ciprofloxacin, ceftriaxone, and *Pseudomonas aeruginosa* was 100% susceptible to both drugs. *Klebsiella species* and *Pseudomonas aeruginosa* showed different resistant pattern for gentamycin and chloramphenicol. *Escherichia colli* was found to be resistant organism from gram-negative.

*Staphylococcus aureus* was the only gram-positive isolate identified, and had sensitivity to ciprofloxacillin and cloxacillin. It was 100 % sensitive to ceftriaxone and gentamycin but found to be resistant to chloramphenicol.

Ciprofloxacin and ceftriaxone had best sensitivity with P-values of 0.021 and 0.001, respectively. Chloramphenicol was found to be resistant to both Gram-positive and Gram-negative organisms (Table 4).

**Table 4:** Organisms’ sensitivity to antibiotics in the surgical ward of WUNEMMH from January 1 to September 1, 2017

Sensitivity	*klebsiella species.*	*Staphylococcus aureus*	*Pseudomonas*	*Escherichia colli*	Total sensitivity	P-value
chloramphenicol (CAF)						
Resistant	2 (33.3)	4 (80)	1 (100)	2 (100)	9	0.124
Sensitivity	4 (66.6)	1 (20)	0	0	5	
gentamycin						
Resistant	3 (33.3)	0	1 (100)	1 (50)	5	0.135
Sensitivity	6 (66.6)	3 (100)	0	1 (50)	10	
ciprofloxacillin						
Resistant	1 (11.1)	1 (25)	0	2 (100)	4	0.021
Sensitivity	8 (88.9)	3 (75)	2 (100)	0	13	
ceftriaxone						
Resistant	1 (11.1)	0	0	2 (100)	3	0.001
Sensitivity	8 (88.9)	3 (100)	2 (100)	0	13	
cloxacillin						
Resistant		1 (25)				
Sensitivity		3 (75)				

### Factors associated with surgical site infection

Surgery waiting time >7 days, Operation time >1 hours, administering AMP before 1 hour of operation, and smokers were factors independently associated with SSIs (Table 5).

**Table 5:** Results of binary and multivariable logistic analysis indicating factors significantly associated with SSI in the surgical ward of WUNEMMH from January 1 to September 1, 2017 (*N* = 255)

Characteristics		Surgical site infection		RR(95% CI)	ARR(95% CI)	*P*-value
		Yes N (%) 42 (16.5%)	No N (%) 213 (83.5%)			
Surgery waiting time	>7 days	33 (55%)	27 (45%)	1.92 (1.12–3.31)	2.48 (1.28–4.79)	0.007*
	<=7 days	9 (4.6%)	186 (95.4%)		1	
Smokers	Yes	18 (30%)	42 (70%)	7.55 (2.50–22.78)	8.01 (2.15–29.84)	0.002*
	No	24 (12.3)	171 (87.7%)		1	
Operation time	>1 h	33 (32.4%)	69 (67.6%)	2.57 (1.56–4.23)	2.13 (1.18–3.86)	0.012*
	<=1 h	9 (5.9%)	144 (94.1%)		1	
Administering antibiotic before 1 h of operation	> 1 h	30 (20%)	120 (80%)	5.27 (2.05–13.54)	5.05 (1.79–14.21)	0.002*
	<=1 h	12 (11.4%)	93 (88.6%)		1	
Residence	Rural	33 (22.4%)	114 (77.6%)	1.57 (0.689–6.786)	1.53 (0.124–3.740)	0.691
	Urban	9 (8.3%)	99 (91.7)		1	
Age in year	1–18	6 (8.7%)	63 (91.3%)	0.79 (0.305–5.346)	1.71 (0.206–8.723)	0.849
	19–40	9 (8.3%)	99 (91.7%)		1	
	> 40	27 (34.6%)	51 (65.4%)	7.11 (1.47–15.022)	7.72 (8.46–40.810)	
ASA Score	>1	39 (20.6%)	150 (79.4%)	6.09 (0.607–29.16)	6.27 (0.52–15.501)	0.076
	<=1	3 (4.5%)	63 (95.5%)		1	
Co-morbidity	Present	6 (28.6%)	15 (71.4%)	3.51 (0.545–8.96)	1.39 (0.154–16.11)	0.358
	Absent	36 (15.4%)	198 (84.6%)		1	
Previous surgery	Yes	12 (40%)	18 (60%)	2.23 (1.540–15.62)	7.64 (0.639–34.04)	0.678
	No	30 (13.3%)	195 (86.7%)		1	
Wound class	Clean-contaminated	27 (24.3%)	84 (75.7%)	1.52 (0.194–1.388)	3.54 (0.70–4.698)	0.185
	Clean	15 (10.4%)	129 (89.6%)		1	

*RR* Relative risk, *ARR* Adjusted relative risk

*statistically significant

Smokers were 8.01 times more likely to develop SSIs compared with non-smokers [ARR = 8.01(95%CI (2.15–29.84)]. Moreover, Hospital stay more than 7 days preoperatively were likely to increase SSI by odd of 2.48 times compared with preoperative hospital stay less than 7 days [ARR = 2.48 (95% CI (1.28-4.79)].

Patients with operation time more than 1 hour increased SSI by 2.13 times compared with patients whose operation finished within 1 hour [ARR = 2.13 (95% CI (1.18-3.86)].

Administering antibiotic before 1 hour were likely to increase SSI by risk of 5.05 times more than those who did not get AMP administration within 1 hour [ARR = 5.05(95% CI(1.79-14.21)] (Table 5).

## Discussion

Out of 255 patients who underwent surgical procedure, 42 patients developed SSIs which contribute to the overall rate of 16.5%. This was consistent with similar studies done in Ethiopia 19.1% [21], Uganda 16.4% [23] and Nigeria 20.3% [24]. But, it is relatively less compared to study done in Tanzania 26% [25]. This difference might be for the reason that our study included only wounds which are clean and clean-contaminated while other studies included contaminated and dirty wound. The finding was higher compared with studies done in USA 7.2% [26] and Saudi Arabia 6.8 % [27]. The difference might be due to modern surgical techniques, surgery rooms and sufficient trained professionals in developed countries.

Smoking contributes to the development of SSI by causing vasoconstriction which results in tissue hypoxia and hypovolemia locally as well as systemically which delays the healing progress, an environment conducive to SSI. In our study patients who smokes were 8.01 times more likely to develop SSIs compared with non-smokers with [ARR = 8.01 (95% CI (2.15-29.84), P= 0.002]. This study is in support of study done in Tanzania [25].

Waiting time for surgery at hospital exposes the patients to contamination or colonization by pathogens which will contribute to the occurrence of SSI [28]. Also patients with long stay in hospital before surgery were sicker or had higher comorbidities that increase their risk for SSI. Hospital stay more than 7 days preoperatively was likely to increase SSIs by risk of 2.48 times compared with preoperative hospital stay less than 7 days, [ARR = 2.48 (95% CI (1.28-4.79), P= 0.007]. Similar to our finding, study done in Egypt showed that patients who underwent operation within 2 days of admission decreased risk of SSI more than those whose operation done after 2 days (P= 0.0034) [29]. This confirms that as the duration of hospital stay preoperatively gets shorten, it minimize rate of SSIs.

In our study, the rate of SSIs were 10.4% and 24.3% in the clean wound and in the clean-contaminated wound respectively. The finding was in contrast with study showed in Tanzania which indicated the rate as clean (63%) and clean-contaminated (33.7%) [25]. As well as in study conducted in Egypt indicated that 57 % and 20% were clean and clean-contaminated wounds respectively [29]. Similar findings have been documented in India where the rate of SSI was higher in clean-contaminated wound (22.8%) compared to wound which are clean (15.1%) [28]. Also in study done in Sudan the rate of SSI was higher in clean contaminated (9.5%) relative to clean wound (8%) [30]. The lesser rate of SSIs in clean wound in our study might be due to close precaution was taken to clean wound and might also be due to rational use of ceftriaxone for the most clean wound, which might result in sensitive strains in the clean wound as indicated [31] and also our study confirms that ceftriaxone is highly sensitive to suspected organisms in clean wound.

The prolonged operation is an independent factor for SSIs in other study because of higher risk for infection due to incision of the operation site [32]. It also increases the extent of tissue trauma due to an extensive surgical procedure, long duration of anesthesia effect, increased blood loss and also decrease surgical antimicrobial prophylaxis concentration at tissue level which all increases the risk of SSIs [25, 28]. In our study, patients with duration of operation more than 1 hour were 2.13 times more likely to develop SSI compared to patients whose operation completed within 1 hour, [ARR = 2.13 (95%CI(1.18-3.86),P= 0.012] which was in line with other study [33].

Administering AMP before 1 hour was observed to be an independent factor associated with SSIs in previous study [34]. In this study, AMP administration before 1 hour increased SSIs risk by 5.05 times compared with administration of first dose of surgical antimicrobial prophylaxis within 1 hour of skin incision [ARR =5.05 (95% CI(1.79-14.21),P=0.002]. Antimicrobials concentration is not sufficient enough at the action site in tissue, as well as in serum to avoid the contamination during operation until wound closure if the first dose of antimicrobial was administered 1 hour earlier. One study indicated that low antibiotic concentration in tissue at the time of wound closure was an independent factor for the development of SSIs [35].

Concerning to the causative agents, common cause of SSI were *Klebsiella pneumoniae* followed by *Staphylococcus aureus, Escherichia colli* and *Pseudomonas aeruginosa*. Most of the operations were caesarean section (CS) and appendectomy which were either clean or clean-contaminated. Our study suggested that the probable reason for the organisms to cause SSI was the spillage of this organism from the GIT. The aerobic-anaerobic organisms closely similar with the normal endogenous flora of the operated organ like clean-contaminated surgery of CS constitutes the most identified bacterial agents.

Gram-negative bacilli are the most common aerobic bacterial agents causing SSIs in CS patients and our finding showed that the predominant etiology was *Klebsiella species*.

The finding of our study is concordant with other studies done Nigeria, Uganda, Tanzania [36–39] which showed that gastrointestinal tract (appendectomy) is a natural habitat for *Klebsiella pneumonia’s*.

In our study resistance to antibiotics were from 11.1% for *Klebsiella* to 100% *(Escherichia colli).* A similar finding has been documented with other studies conducted in Mbarara, Mulago and Tanzania [37–39].

In our study *Klebsiella pneumoniae* has high sensitivity to both ciprofloxacin and ceftriaxone but was resistant to ampicillin, tetracycline, and erythromycin. In line with our study, study conducted in Kenya showed that *Klebsiella's* resistant to ceftriaxone [40] and resistance to chloramphenicol was also reported in Uganda [41].

Our study showed that ciprofloxacin, ceftriaxone, cloxacillin, and gentamycin were sensitive to *Staphylococcus aureus,* but were resistant to erythromycin, chloramphenicol, and ampicillin. This was in line with a study done in Uganda [41]. The finding for ceftriaxone in our study is surprisingly indicated high sensitivity against both Gram-negative and Gram-positive organisms since the drug is frequently prescribed to almost all patients who underwent operation.

The result of this study will provide information for surgeons, working organizations like Non-governmental organizations (NGOs) in health care system of WUNEMMH as well as in national and international level to decrease the rate of SSI and related complications with SSIs.

## Limitation

Professionals’ related variables, antiseptics used, surgical equipment sterilization methods and anesthesia type were not included due to resources shortage.

Despite limitation, the study provides baseline information on rate of SSI, associated factors of surgical site infection as well as on the causative agents and their antimicrobial susceptibility in the surgical ward of WUNEMMH.

## Conclusion

The rate of SSIs was 16.5% which was lower compared with some developing countries and higher relative to reports from developed countries. Klebsiella pneumonia found to cause the majority of SSI. Ceftriaxone and ciprofloxacin were sensitive antibiotics.

Surgery waiting time more than 7 days, smokers, administrating antibiotic before 1 hour and operation time more than 1 hour were independently associated with SSIs.

Antimicrobial resistance is one of world problem, which results partly due to unnecessarily administration of antibiotic for long period so administration of surgical AMP for long time should be avoided.

It becomes imperative, therefore, to know local antibiotic sensitivity patterns existing in a hospital to plan appropriate antibiotic policy.

In addition, periodic surveillance on rate of SSI, associated factors of surgical site infection, causative agents, and their antimicrobial susceptibility will decrease the rate.

A surveillance system for SSI with feedback of proper data to surgeons and hospital authorities is highly recommended to reduce SSI rate in WUNEMMH.
